# Adaptive plasticity and epigenetic variation in response to warming in an Alpine plant

**DOI:** 10.1002/ece3.1329

**Published:** 2015-01-13

**Authors:** Adrienne B Nicotra, Deborah L Segal, Gemma L Hoyle, Aaron W Schrey, Koen J F Verhoeven, Christina L Richards

**Affiliations:** 1 Research School of Biology, The Australian National UniversityCanberra, Australian Capital Territory, Australia; 2 Department Biology, Science Center, Armstrong University11935 Abercorn Street, Savannah, Georgia, 31419; 3 Department of Terrestrial Ecology, Netherlands Institute of Ecology (NIOO-KNAW)Droevendaalsesteeg 10, Wageningen, 6708 PB, the Netherlands; 4 Department of Integrative Biology, University of South FloridaTampa, Florida, 33617

**Keywords:** Adaptive plasticity, amplified fragment length polymorphism, alpine plants, DNA methylation, epigenetics, global warming

## Abstract

Environmentally induced phenotypic plasticity may be a critical component of response to changing environments. We examined local differentiation and adaptive phenotypic plasticity in response to elevated temperature in half-sib lines collected across an elevation gradient for the alpine herb, *Wahlenbergia ceracea*. Using Amplified Fragment Length Polymorphism (AFLP), we found low but significant genetic differentiation between low- and high-elevation seedlings, and seedlings originating from low elevations grew faster and showed stronger temperature responses (more plasticity) than those from medium and high elevations. Furthermore, plasticity was more often adaptive for plants of low-elevation origin and maladaptive for plants of high elevation. With methylation sensitive-AFLP (MS-AFLP), we revealed an increase in epigenetic variation in response to temperature in low-elevation seedlings. Although we did not find significant direct correlations between MS-AFLP loci and phenotypes, our results demonstrate that adaptive plasticity in temperature response to warming varies over fine spatial scales and suggest the involvement of epigenetic mechanisms in this response.

## Introduction

Climate change is altering the environments in which all organisms develop. Plant species can respond to these novel conditions through phenotypic plasticity, adapt through natural selection, or migrate to follow conditions to which they are adapted. The amount of variation in natural populations for traits that will be critical in future climates is generally unknown (Davis and Shaw 2001; Parmesan 2006) and understanding trait response, both phenotypic and ultimately genetic, will be critical for predicting how organisms will respond to novel abiotic conditions (Parmesan 2006). Predicting these responses to environmental changes will require an understanding of the environmentally induced variation in the phenotype of individuals (phenotypic plasticity) and the molecular mechanisms underlying those responses (Nicotra et al. 2010).

Some portion of plastic variation may be adaptive, and some is likely to be neutral or even maladaptive (van Kleunen and Fischer 2005). Of particular interest when predicting the response of species to climate change is the portion of the reaction norm that reflects active, adaptive plasticity that leads to an increase in mean global fitness for the genotype (see Nicotra et al. 2010 and references therein). Selection analyses, in which fitness is regressed against trait plasticity, provide a tool to assess the adaptive value of a plastic response (van Kleunen et al. 2000; Stinchcombe et al. 2004).

Adaptive plasticity is predicted to evolve when a species is subjected to fine-scale environmental heterogeneity relevant within the life span of the organism and when conditions can be predicted based on environmental cues (Sultan et al. 1998; Valladares et al. 2000, 2002; Herman et al. 2014). Thus, species that are distributed across strong environmental gradients present an ideal system in which to examine drivers and consequences of phenotypic variation, particularly in a climate change context.

Considering predicted shifts in temperature and precipitation (IPCC 2007), alpine systems provide a unique opportunity to explore the importance of plasticity in response to global climate change. Alpine systems have steep temperature and water availability gradients associated with elevation, local topography, and aspect. Within-species variation in trait means has been observed in several alpine species. For example, specific leaf area (SLA) and leaf length decrease (Korner et al. 1986; Byars et al. 2007; Garibaldi et al. 2011; Zhang et al. 2012), height decreases (Wang et al. 2008; Hoffmann et al. 2009) and seed mass increases (see references in Segal 2010) with increasing elevation. Exploring the mechanisms that underlie such phenotypic patterns will provide a better understanding of the capacity of alpine plants to respond to future climates.

Recent studies suggest that phenotypic plasticity can be mediated through epigenetic effects (Richards et al. 2008; Bossdorf et al. 2010; Scoville et al. 2011; Herrera and Bazaga 2013; Zhang et al. 2013; Herman et al. 2014). The most studied epigenetic effect is DNA methylation which has been shown to increase in variance in response to stressful conditions (Verhoeven et al. 2010; Dowen et al. 2012) and has known effects on ecologically important phenotypes (Johannes et al. 2009; Bossdorf et al. 2010; Zhang et al. 2013; Cortijo et al. 2014). Because epigenetic states can be thus altered, epigenetic effects could provide a rapid source of phenotypic variation without any change in genetic variation (Rapp and Wendel 2005; Bossdorf et al. 2008), thereby affecting the ability of populations to persist in the face of changing climate. Attempts to probe this connection between epigenetic mechanisms and phenotypic responses have thus far been limited.

We examined the extent and correlates of adaptive phenotypic plasticity in response to growth temperature in half-sib lines collected across an elevation gradient for the alpine herb, *Wahlenbergia ceracea* Lothian (Campanulaceae). We asked whether seed that had developed at low, intermediate, and high elevation within the species' natural range differed in genetic structure, mean trait values, or in plasticity in response to temperature. In addition, we assessed variation in DNA methylation between maternal plants and offspring and between offspring from different elevations grown under different temperature regimes to assess the presence of heritable or induced variation in epigenetic markers. The results provide striking evidence of differentiation in trait means and plasticity over small geographic distances and suggest that adaptive plasticity is associated with increased variation in DNA methylation.

## Material and Methods

### Study species and seed collection

*Wahlenbergia ceracea* Lothian (Campanulaceae) is an alpine short-lived perennial herb found in moist sites in high montane and alpine elevations in Australia (∽1600–2200 m). We collected mature seed capsules from five plants at each of three elevation ranges: low (1610–1625 m asl), medium (1750–1830 m asl), and high (1940–1975 m asl) in Kosciuszko National Park, NSW, Australia (−36.432, 148.338), relative to the natural distribution of the species in the park. Higher elevation sites are on average colder, have longer periods under snow and experience fewer extreme heat and freezing events (Briceño Rodriguez 2014). Plants were a minimum of 10 m apart and the total range between any two plants was 14 km (Appendix A1). A related study showed that plants were significantly shorter at higher elevations but showed few other morphological differences under field conditions at maturity (Segal et al. unpubl results). Seed were collected at the point of natural dispersal in 2011 to yield 15 half-sib maternal family lines. We found no correlation between elevation and seed mass (Segal 2010).

### Seedling growth conditions

We planted three seeds from each of the 15 maternal lines into each of ten 50 × 123 mm (210 mL) tubes using commercial seed-raising mix (Debco Pty Ltd, Victoria, Australia) with micronutrients (December 20–21, 2011). Tubes were randomly assigned to one of five blocks so that each block contained two replicates from each line, one per temperature treatment. We grew five blocks each in two controlled temperature glasshouses to test the effect of a cool (mean ± SD of 20.6 ± 0.02°C/11.5 ± 0.01°C day/night temperature on a 12 h cycle) and a warm (mean ± SD of 29.8 ± 0.02°C/19.2 ± 0.02°C day/night) temperature regime on seedling growth and development. We arranged tubes randomly within blocks on the glasshouse bench and matched block locations between the adjacent warm and cool temperature glasshouse chambers to minimize differences in light and air circulation. Overall soil temperatures (measured with iButton (Maxim Integrated, San Jose, CA) loggers in soil trays) were ∽4°C higher in the warm than the cool glasshouse both day and night (Hoyle et al. 2013). The cool glasshouse roughly approximated mid-day early growing season soil temperatures in situ and the warm glasshouse temperature regime represented current summer high temperatures (Hoyle et al. 2013), although without the variation present in situ (see also Hoyle et al. 2013; Briceño et al. 2014).

We recorded date of seedling emergence and pinched out all but the largest seedling in each tube before seedlings had begun to shade one another. We measured height (mm, to top of shoot apical meristem or base of bolt on flowering plants), rosette diameter (length across two opposite leaves at widest point), and number of true leaves 8, 11, and 14 weeks after sowing as well as 90 days after germination. Date of first flowering was recorded for each seedling that flowered in the first 20 weeks of the experiment after which time data were recorded monthly. We monitored total capsule production until plants naturally senesced.

Hand pollination trials revealed no significant effect of hand pollination on either seed number per capsule or individual seed mass. Thus, we conclude that *W. ceracea* is autogamous and that capsule and seed production in the glasshouse is not likely to be limited by pollen (A2).

### AFLP genotyping and MS-AFLP epi-genotyping

We collected leaf tissue from the high-elevation and low-elevation maternal plants in the field, dried it in a plant press and froze at −20°C. From each of three or four offspring per maternal line grown under each of the controlled conditions, we collected and immediately froze leaf tissue (−20°C). For both generations, tissue was collected at the peak of the growing season and before senescence. We screened a total of five high-elevation and five low-elevation maternal plants and 66 offspring individuals for genetic variation using AFLP (*N* = 76). We used the Qiagen DNeasy Plant Mini kit (Qiagen, Valencia, CA) to perform duplicate DNA extractions from each sample and ran the duplicates through the AFLP protocol to ensure reliable scoring of fragments. The standard protocol suggested by Qiagen was used, eluting the DNA with water instead of TE in the final step. The AFLP protocol was based on standard methods with some modifications (Richards et al. 2008, 2012). In the selective amplification, we multiplexed two primer pairs using 4 pmol of the 6-carboxy-fluorescein (6-FAM) fluorescently labeled *EcoRI*+AGC primer (/56-FAM/TACTGCGTACCAATTCAGC) with 4 pmol of the 4,7,2′,4′,5′,7′-hexachloro-6-carboxyfluorescein (HEX) fluorescently labeled EcoRI+ACG primer (/5HEX/TACTGCGTACCAATTCACG) and 25 pmol of the MseI+CAA in standard selective amplification reaction mixture and PCR conditions (Richards et al. 2008).

We visually inspected the AFLP fragments using the open source program PEAKSCANNER v 1.0 (Applied Biosystems) and manually scored approximately 200 total loci for the HEX and 6FAM primer sets combined with a binary code, zero for band absent, one for band present. Of the 200 loci identified, nine were polymorphic. The repeatability of banding patterns across duplicate samples was assessed to determine whether banding patterns were consistent, only positions that could be reliably scored were included in the analysis. Throughout, we use “locus” to indicate a specific fragment size in the AFLP and MS-AFLP results. We use “haplotype” to indicate the collection of binary variable positions (dominant genotypes) for each individual at AFLP loci, and “epi-genotype” to indicate the collection of binary variable positions at MS-AFLP loci.

We screened the 76 individuals for epigenetic variation with MS-AFLP using the same duplicate DNA extractions used for AFLP and the same selective bases on the two *EcoRI*+3 primers multiplexed with 25 pmol of the *HpaII/MspI*+TCAC primer (ATCATGAGTCCTGCTCGGTCAC). The MS-AFLP analysis used essentially the same protocol as the AFLP, but the *Mse*I enzyme was replaced with the same concentration of either *Msp*I or *Hpa*II, both of which are methylation sensitive, but vary in sensitivity. Both enzymes recognize and cleave CCGG sequences, but cleaving by *Hpa*II is blocked when the inner or outer C is methylated at both strands, while cleaving in *Msp*I is blocked when the outer cytosines are fully or hemi-methylated; cleaving in both enzymes is blocked when both cytosines are methylated. Four different types of variation can be scored (Salmon et al. 2008): Type I if both enzymes cut at the restriction site (no methylation), Type II if HpaII does not cut and MspI does cut (restriction site has a methylated internal C), Type III if HpaII does cut and MspI does not (restriction site has a methylated outer C), and Type IV if neither enzymes cuts (either both Cs are methylated or the restriction site has mutated). Recent work indicates that type II and III variation cannot be simply interpreted as CG versus CHG methylation, because what looks like CHG methylation is in fact often caused by differently methylated internal restriction sites nested within fragments (Fulnecek and Kovarik 2014). Therefore, we pooled data into two categories, methylated (Type II, Type III) or not methylated (Type I) restriction sites. We treated Type IV as missing data, because the methylation state cannot be specified (Salmon et al. 2008). We identified approximately 150 loci that could be reliably scored for *Msp*I and *Hpa*II for the HEX and 6FAM primer sets combined. Of these 150 loci, 39 were polymorphic.

### Statistical analysis

#### Phenotypic analyses

We used REML models in Genstat (VSN International, 14th edn) with block, growth temperature (cool vs. warm chamber), and elevation (low, medium, or high) as fixed effects to analyze phenotypic data. We assessed maternal line effects by nesting families within elevation. Initial analyses included a continuous variable and a square term for elevation to account for nonlinear effects, but as this did not improve model fit, the term was not applied in the final model. Data were log transformed as needed to meet assumptions of normality.

We calculated a modified Plasticity Index (PI) to compare cool and warm treatment values for each maternal line in each block (after Valladares et al. 2006). We calculated the plasticity index as (maximum − minimum)/maximum of the ln-transformed values for each pair of plants within a block*maternal line combination (*n* = 5 pairs for each line). Pairs in which both individuals survived but neither set fruit (zero fitness), and pairs in which one plant died were excluded from the plasticity analysis (a total of 13 of the 75 pairs).

Selection gradient analysis was performed to determine whether plasticity was adaptive, maladaptive, or neutral. The plasticity index was standardized to a mean of zero and standard deviation of one. Relative fitness was calculated as the mean fitness (capsule number) for a pair of plants divided by mean of all pairs and then log transformed (van Kleunen and Fischer 2001). The linear model included a term for the ln-transformed trait mean for each pair of plants (standardized as for PI), block, elevation, and maternal family nested within elevation. By pairing plants across blocks for our PI calculation, we were able to account for variation in plasticity within maternal line; conventional approaches that calculate plasticity by taking averages across plants for a given line within treatments cannot incorporate that variation. Preliminary analyses demonstrated strong interactions between measures of plasticity and elevation; therefore, plasticity was assessed as the interaction between elevation and PI for the trait. The partial regression coefficients for each elevation were used to assess slope and significance of the selection differentials.

#### Genetic analysis: AFLP

GENALEX version 6.41 (Peakall & Smouse 2006) was used to identify shared haplotypes among individuals and to determine the haplotype diversity (*h*-AFLP) for each sample. We compared *h*-AFLP between maternal plants and seedlings, and among four categories of seedlings: high-elevation mother grown cool, high-elevation mother in warm, low-elevation mother in cool, and low-elevation mother in warm.

We also used GENALEX to calculate estimates of genetic differentiation between samples over all loci using an AMOVA framework. AMOVA was used first to compare variances among all individuals from either high or low elevation (Φ_ST_) and then to compare only seedlings between high and low elevation. Finally, we performed an AMOVA to compare variances among seedling treatments, both over all treatments and pairwise between treatments. For all AMOVA analyses, 9999 permutations were calculated to estimate statistical significance and the initial alpha = 0.05.

#### Epigenetic analysis: MS-AFLP

We analyzed epigenetic variation among individuals from the two elevations in the two temperature treatments. Statistical methods followed those in the genetic analysis. Given the small sample size for maternal plants and the differences in growing conditions, we appreciate that the epigenetic results for the mothers can only be compared loosely to the offspring but include these analyses as it is still interesting to assess the behavior of the markers.

To further explore effects of experimental temperature treatment on MS-AFLP profiles, we compared the proportion of methylated loci (types II and III, see above) among all scorable loci (types I, II and III) between plants using a generalized linear model with a binomial distribution and logit link function (GENMOD procedure in SAS 9.2, The SAS Institute, Cary, NC). We tested effects of elevation, temperature treatment, maternal line (nested within elevation), temperature treatment x elevation, and temperature treatment x maternal line, on the proportion of methylated loci. To correct for overdispersion, standard errors were scaled using the Pearson chi-square (pscale option in GENMOD). Based on multivariate analysis of the MS-AFLP profiles, we tested for differences in multivariate dispersal between warm-grown and cool-grown plants using the PERMDISP program (Anderson et al. 2006; a multivariate analogue of the Levene's homogeneity of variances test). For this purpose, an epigenetic dissimilarity matrix was derived based on simple matching coefficients using the DISTANCE procedure in SAS 9.2. With this distance measure, shared methylations (type II or III fragments) and shared nonmethylations (type I fragments) at polymorphic MS-AFLP loci contribute equally to the similarity score between two individuals; both can capture relevant epigenetic information (Schulz et al. 2013).

Mantel tests were used to test for correlations between epigenetic and phenotypic data. We made a trait-based Mahalanobis distance matrix for the experimental seedlings using all available measurements on plant height, rosette diameter, leaf number, flowering time, and capsule production (see section “seedling growth conditions”). Mahalanobis distances can handle variables that are correlated and/or measured at different scales by first extracting standardized principal components from the set of variables and calculating pairwise Euclidean distances based on these principal component scores (PRINCOMP and DISTANCE procedures in SAS 9.2). We ran mantel tests using Zt software (van de Peer 2002).

## Results

### Effects of temperature on growth and reproductive traits

Seedlings in the cool temperature regime took longer to emerge than those in the warm regime; this pattern was not affected by the elevation at which the seed were developed (Appendix A3, *P* ≤ 0.0001). In addition, maternal lines differed in time to emergence and this variation was not dependent on growth temperature or elevation (Appendix A4, *P* ≤ 0.019).

Not surprisingly, seedlings from the warm glasshouse, which had emerged earlier, were generally larger (taller with greater rosette diameter and more leaves) than those in the cool glasshouse when compared at constant times after sowing (for *P* values see Appendix A4, Fig.A–C). In addition, warm-grown seedlings showed higher relative growth increments in the juvenile phase, although the extent of this effect varied among blocks (Appendix A4). Even when compared at a standard time postgermination (90 days), warm-grown plants were on average taller with broader rosettes (Appendix A4, *P* ≤ 0.002, 0.003, respectively). With the exception of height growth increment at 90 days, there was no significant variation among maternal lines in growth parameters.

**Figure 1:**
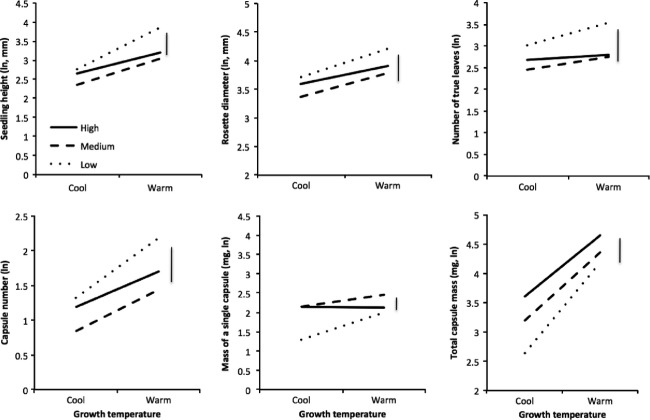
Measurements of (A) seedling height, (B) rosette diameter, and (C) number of true leaves at 14 weeks post sowing and (D) total capsule production, (E) mass of a single capsule, and (F) total capsule mass. Vertical bar indicates least significance difference among means.

Plants in the warm regime also flowered earlier and produced more capsules than those in the cool room (Fig.D, *P* ≤ 0.001, Appendix A3). In addition, capsules were larger under warm conditions (Fig.E, *P* ≤ 0.004, Appendix A3). When we estimated total mass of capsules from individual capsule mass and capsule number (for those plants on which mass was determined, see Appendix A2), we found that total capsule mass was also greater at warm temperatures (Fig.F, *P* ≤ 0.001, Appendix A3).

More striking, however, was the elevation effects: seedlings from seed that had developed at lower elevations grew taller had greater rosette diameter and had more leaves than seedlings grown from seed that had developed at high elevations (Fig.A–C, Appendix A4). Seedlings from mid-elevations were generally not significantly different from high-elevation seedlings. The effect of elevation was significant for all growth measurements at each of the three measurement times except for rosette diameter at 14 weeks (Appendix A4). Note, however, that the elevation effects were not significant at 90 days postgermination, nor were they seen in the relative growth increments. Plants of low-elevation origin also produced more capsules than those from higher elevations (Appendix A3 you, *P* ≤ 0.004, Fig.1D), but individual capsules were larger on plants from high-elevation families (Fig.1E), and so on average, seedlings from low-elevation families had lower total capsule mass (Fig1F).

### Selection gradient analysis of plasticity

**Figure 2:**
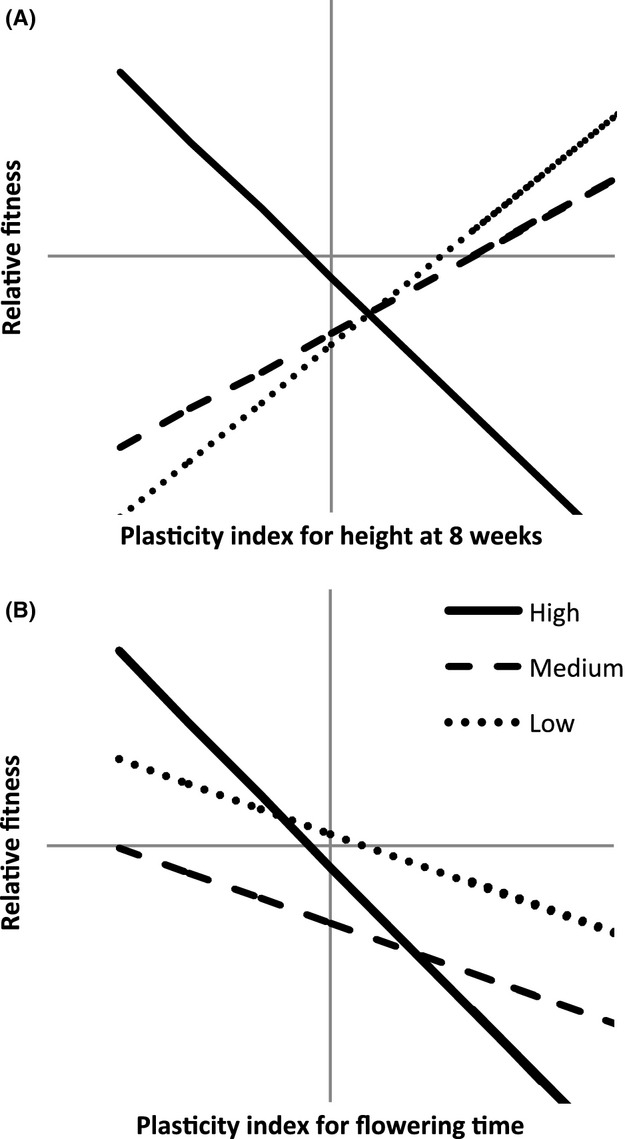
Example selection gradient analysis plots showing significant relationships between the fitness proxy (capsule number) and plasticity in (A) height at 8 weeks and (B) flowering time.

Selection gradient analysis demonstrated a pattern of adaptive plasticity that was in accord with the previous results: In general, the more plastic low-elevation families also showed adaptive plasticity, whereas the less plastic high-elevation families were more likely to exhibit negative selection differentials for plasticity (Table1, Fig.2A). Mid-elevation families were intermediate in trait values and plasticity measures, and for these, plasticity was generally neutral. This pattern was observed to varying extents in several traits. Notably, one trait showed negative selection differentials for plasticity at all elevations: flowering time. Earlier flowering was associated with greater capsule production in all treatments (Fig.2B, Table1).

**Table 1:** Selection gradient analysis for trait means and plasticity. (A) ANOVA table for analysis of effects of trait means and plasticity on relative fitness. Only traits with significant selection gradients for plasticity are included. Note that the covariate mean was also significant for height at 11 and 14 weeks and 90 days, leaf number at 90 days, and diameter at 8 and 14 weeks, but plasticity index was not. (B) Selection differentials (β) on plasticity at each elevation and across elevations. Underline text and negative numbers indicate negative selection differentials (costs); bold text indicates significant slopes (differentials, at *P* < 0.05). Figures in italics are significant at *P* = 0.1

	df	Height 8 weeks	Juv. height growth	Rosette diam 90 days	Leaf no. at 11 weeks	Leaf no. at 14 weeks	Days to flowering
(A) Analysis of variance table							
Trait mean	1	**<0.001**	0.156	<0.001	**<0.001**	**<0.001**	**0.007**
Block (df = 4)	4	**0.027**	0.341	0.646	0.435	0.682	0.086
Elevation	2	0.565	0.455	0.67	0.651	0.818	0.267
Elevation. family	12	0.340	0.292	0.559	0.311	0.482	0.244
Trait *P* × elevation	3	**0.015**	*0.073*	0.064	*0.057*	0.103	**0.010**
(B) Selection differential, β							
Mean		0.311**	−0.050	0.515**	0.332*	*0.268*	−0.281*
High		−0.498*	−0.390*	−0.118	−0.081	0.006	−0.559**
Medium		0.153	−0.157	0.203	−0.107	0.311	−0.196
Low		0.393	0.278	0.502*	0.452**	0.536*	−0.194

*P < 0.05, **P < 0.01.

### Genetic diversity and structure

**Figure 3:**
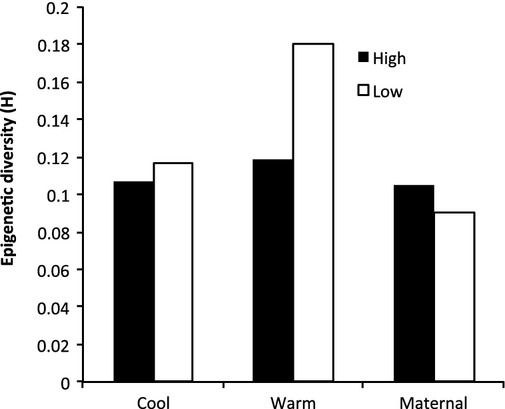
Comparison of genetic haplotype diversity (h-AFLP) and epigenetic haplotype diversity (h-MS-AFLP) among samples of *Wahlenbergia ceracea*. Samples are all maternal plants (Maternal), maternal plants from low elevation (M-L), maternal plants from high elevation (M-H), all seedlings (Seedling), seedlings from low altitude mothers in warm treatment (S-Lw) and in cool treatment (S-Lc), and seedlings from high altitude mothers in warm treatment (S-Hw) and in cool treatment (S-Hc).

There were only nine variable positions among individuals from approximately 200 loci across the *Mse-I* AFLP selective PCR products, which formed 20 haplotypes. Haplotype diversity (*h*-AFLP) was similar between mothers and seedlings (*h*-AFLP = 0.019), while seedlings from low-elevation mothers had slightly, but significantly higher diversity than seedlings from high-elevation mothers (*P *≤* *0.05, Fig.3, Appendix A4). Likewise, there was little, but significant, genetic differentiation between individuals from low and high elevation among all individuals (Φ_ST_ = 0.027, *P *=* *0.047) and among only seedlings (Φ_ST_ = 0.038, *P *=* *0.027). There was no significant genetic differentiation among seedling treatments (Φ_ST_ = 0.022, *P *=* *0.133) or between any pairwise comparison of seedling treatments.

### Differences in epi-genotype between generations and as a function of elevation and growth temperature

Our analysis revealed more epigenetic than genetic diversity (more than four times as many variable positions), and the offspring had more epigenetic diversity than maternal plants. Thirty-nine variable positions were detected among 150 loci observed, which formed 62 epi-genotypes. There was no significant epigenetic differentiation between individuals from low and high elevation among all individuals (Φ_ST_ = 0.011, *P *=* *0.09) or among only seedlings (Φ_ST_ = 0.013, *P *=* *0.082). Nor was there significant epigenetic differentiation among seedling treatments (Φ_ST_ = 0.0001, *P *=* *0.475) or between any pairwise comparison of seedling treatments. In addition, there was no significant differentiation between maternal plants from low- and high-elevation sites, but note that sample size was very small for this comparison (*n* = 5 from each elevation, *F*_st_ 0.01, *P* = 0.05).

Haplotype diversity for epi-genotypes (*h*-MS-AFLP), however, was higher in seedlings (*h*-MS-AFLP = 0.115) compared to maternal plants (*h*-MS-AFLP = 0.094). High-elevation maternal plants had slightly higher diversity (*h*-MS-AFLP = 0.099) compared to low-elevation maternal plants (*h*-MS-AFLP = 0.089). Notably, there was also significantly greater epigenetic diversity in seedlings from low-elevation origin when grown at warm temperatures (*h*-MS-AFLP = 0.158) compared to low-elevation maternal plants, the cool-grown low-elevation seedlings (*h*-MS-AFLP = 0.109), or the high-elevation origin maternal plants and seedlings (*h*-MS-AFLP = 0.098 & 0.093; Fig.3). Within the high-elevation seedlings, there was slightly more epigenetic diversity in the warm grown than the cool-grown plants, but the difference was not as strong as for low-elevation plants (Fig.3).

Consistent with the difference in epigenetic haplotype diversity between warm-grown and cool-grown plants from low elevations, multivariate dispersal in MS-AFLP profiles based on pairwise distances was significantly larger within the low-elevation warm treatment than in the low-elevation cool treatment (average distance to centroid: 0.20 for cool and 0.27 for warm treatments, respectively; *P* = 0.037). We did not see this difference in multivariate dispersal between warm and cool treatments in the high-elevation seedlings (*P* = 0.87). In summary, although we did not detect differentiation (analogous to mean position in multivariate space) in response to elevation or treatment, we did find a difference in the amount of epigenetic variance within-groups in response to treatment.

Mantel tests detected no correlations between phenotypic variation and epigenetic variation, either for high- (*n* = 31 plants from warm and cool treatments, *r* = −0.04, *P* = 0.40) or low-elevation plants (*n* = 33, *r* = 0.03, *P* = 0.42). These MS-AFLP–phenotype correlation tests were not appreciably affected by first accounting for possible correlations between MS-AFLP variation and temperature treatments (using partial mantel tests that test the MS-AFLP–phenotype correlation after controlling for a design matrix that codes plants from different treatments as distance = 1 and plants from the same treatment as distance = 0; data not shown). This suggests that global phenotypic variation and global MS-AFLP variation do not show consistent association in the experimental data, either within temperature environments or in the response to the temperature difference.

## Discussion

We set out to determine whether the distribution of adaptive plasticity in response to warming conditions differed depending on the elevation at which seed had developed in the alpine herb *W. ceracea*. Further, we assessed genetic and epigenetic differentiation between elevations and asked whether parents or offspring differed in patterns of DNA methylation depending on site of origin or experimental temperature regime. Warming did elicit a plastic response, accelerating seedling emergence and growth and leading to a shorter time to flowering and larger, more numerous capsules. More strikingly, although the maternal plants from which seed were collected were distributed over a very small geographic range, plants were differentiated at AFLP loci and the offspring showed evidence of significant trait differentiation along an elevation gradient. In addition to the genetic differentiation, plants grown from seed developed at lower elevation were larger, produced more seed capsules, and generally showed stronger plastic responses to temperature than those grown from high-elevation plants. The patterns of adaptive plasticity reflect our results for differentiation in plasticity: Plasticity was more likely to be selected for in the more plastic low-elevation seedlings. Finally, seedlings from low-elevation origin also showed the greatest propensity for changes in epigenetic marks in response to growth temperature. These results are consistent with the hypothesis that extreme but less variable conditions at high-elevation sites have led to canalization of growth traits, potentially via suppression of epigenetic change. Plants in lower elevation sites, in contrast, are perhaps likely to be exposed to more frequent extreme heat/cold events and have greater probability of encountering good conditions (e.g., periodic long, warm seasons, Briceño et al. 2014). Thus, the ability to respond to warming temperatures with vigorous growth has obvious value for low elevation.

These results raise several interesting questions: How common is such small-scale differentiation in the distribution of adaptive plasticity, or the propensity for changes in methylation pattern? And, what does the association between epigenetic variation and adaptive plasticity tell us about the mechanisms underlying expression of phenotypic plasticity?

Similar results of small-scale variation in selection for plasticity have been indicated for plasticity in leaf length and rosette circumference in alpine *Poa hiemata*, as indicated by patterns of co-gradient selection (Byars et al. 2007). Also, an altitudinal pattern of plant development persisted in a common garden environment in *Stylidium armeria* (Hoffmann et al. 2009), although this pattern was weaker than in the field, suggesting phenotypic plasticity in combination with local adaptation may contribute to survival of *Stylidium* in the field. Previous work has shown changes in the adaptive value of phenotypic plasticity depending on growth conditions or between sister species or disjunct populations (Jacobs and Latimer 2012). However, the present study is the first we know of to demonstrate contrasting selection gradients on plasticity over such a limited geographic range.

In addition to being more plastic, the low-elevation plants showed a greater propensity to alter epigenetic signature in response to warming. Does this result indicate epigenetics-mediated adaptive phenotypic plasticity that might contribute to a bet-hedging strategy (Herman et al. 2014)? Or might the epigenetic result be an artifact of the genetic differentiation (small though it was) between low- and high-elevation plants, as has been found in previous work (Joseph and Moritz 1993; Herrera and Bazaga 2010; Lira-Medeiros et al. 2010)? Because the epigenetic difference was only apparent at high temperature, we think it is not simply a correlate of genetic differentiation. Although we did not find a majority response at the same loci (i.e., overall genome-wide pattern of differentiation), we did find a general tendency to change across several loci, which differed among maternal lines and elevations. Thus, we argue the evidence is more in favor of a link between epigenetic expression and the adaptive plastic response.

To some extent, the ambiguity may reflect limitations of our method. The different maternal lines are also different genotypes and therefore different fragments across the individuals could be related to similar function. The MS-AFLP approach emphasized genome-wide patterns of variation, while epigenetically mediated functional responses may be restricted to a few specific loci. While this is a known limitation of the AFLP protocol, the approach does allow us to examine epigenetic response in this nonmodel plant for which the genome has not been sequenced (Schrey et al. 2013).

Other studies examining epigenetic signatures have shown increases in variance in response to exposure to different environmental factors (Verhoeven et al. 2010; Dowen et al. 2012), with known effects on ecologically important phenotypes (Cubas et al. 1999; Johannes et al. 2009; Bossdorf et al. 2010; Zhang et al. 2013; Cortijo et al. 2014). Multigeneration experiments have shown that parental exposure to biotic or abiotic stresses resulted in modified DNA methylation in unexposed offspring (Boyko et al. 2010; Verhoeven et al. 2010). In dandelion, MS-AFLP showed that plants with identical genotypes exposed to different stresses had up to 30% change in polymorphic methylation sensitive markers compared to controls (Verhoeven et al. 2010). Bilichak et al. (2012) showed that progeny of plants exposed to salt stress were globally hypomethylated, but hypermethylated at histone lysine methyltransferase genes. In response to water stress, Juenger et al. (2010) found increased expression of several genes related to chromatin or epigenetic regulation. In our data, the larger variation in the low-elevation warm-grown seedlings may be due either to induced random modifications or may arise because different genotypes respond differently to the same environment.

We went a step further than just documenting changes in epigenetic marks in that we examined correlations between these changes, changes in ecologically important traits, and adaptive plasticity therein. While our analysis is limited in scope, both in terms of sample size and the number of markers identified in the epigenetic analysis, we found that the pattern of adaptive plastic responses and propensity for epigenetic change occur within the same individuals (low-elevation progeny, especially when grown under warm conditions). We note, however, that our data did not reveal evidence for direct correlations between MS-AFLP variation and trait variation. Thus, based on these data the functional explanation of the MS-AFLP response remains unclear. The results here indicate adaptive plasticity in a range of complex quantitative traits, each of which is likely to be controlled by a range of genetic pathways, some potentially shared.

The question of what the molecular mechanisms underlying plasticity might be is an old one, and only recently have we begun to unravel the answers. For some systems, the pathways of signal perception and response are well understood, for example, flowering time in *Arabidopsis thaliana* (Mouradov et al. 2002; Simpson and Dean 2002), but these are the minority. MS-AFLPs are a cost effective way to obtain data on methylation marks in many individuals and in nonmodel species and provide genome-wide patterns at anonymous loci. However, truly understanding the role of epigenetics requires a more powerful coverage of the genome and characterization of the behavior of specific genes or regulatory elements (e.g., *A. thaliana*, Slotkin and Martienssen 2007; Lippman et al. 2004). Genetic screening using AFLPs is rapidly being replaced by genotyping by sequencing (GBS) and restriction site-associated DNA sequencing (RAD-seq) approaches in nonmodel species (Narum et al. 2013). As has happened in model species, eventually these approaches will expand to DNA methylation profiling (e.g., reduced representation bisulphite sequencing or RRBS, Boyle et al. 2012), which will provide a powerful tool to explore how methylation patterns behave across different genomic regions in natural environments and will allow for more fine-scale resolution of sequence polymorphisms that we may have missed using AFLP markers.

## Conclusion

In conclusion, our study demonstrates significant differentiation in adaptive plasticity and in patterns of epigenetic expression within a species over a small geographic range. This variation may impact on the potential of the species to tolerate a warming climate. The AFLP analysis indicated only very little differentiation between the low- and high-elevation plants, suggesting that gene flow is quite high across the species range, however, the epigenetic and plasticity results indicate that some genotypes may have a higher potential to respond to stressful conditions which could be critical to surviving future climates.

## Figures and Tables

**Figure A1:**
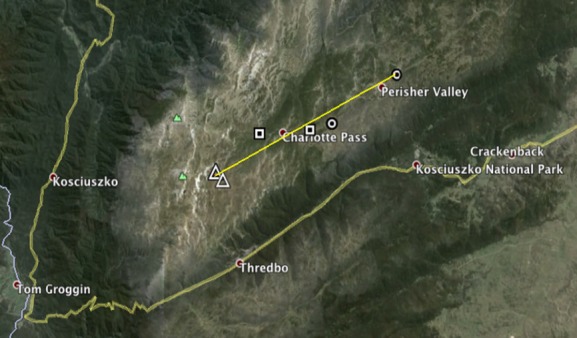
Map of seed collection points. Each point includes 2–3 plants selected at least 10 m apart. High, medium, and low-elevation sites are indicated by triangles, squares, and circles, respectively. The length of the yellow line is approximately 14 km. Red dots indicate other local landmarks including the peak of Mt Kosciuszko, the highest point in Australia.

## Data Accessibility

MS-AFLP and phenotype data: DRYAD entry doi:10.5061/dryad.73r1b.

## Appendix A2

Assessing Autogamy in *Wahlenbergia ceracea*

To determine whether capsule production in the glasshouse was a good indicator of fitness, we assessed whether *Wahlenbergia ceracea* is autogamous (capable of self-pollination). Some *Wahlenbergia* species are autogamous (Mouradov et al. 2002; Simpson and Dean 2002); we assessed whether this is the case in *W. ceracea* by conducting a hand pollination experiment on a subset of plants. Hand pollination of flowers was conducted on no more than half the flowers on a given plant when the stigma was exposed and glossy in appearance. Mature pollen was moved from the subtending anthers to the stigma using a toothpick. Seed mass and number were assessed for capsules from 32 plants comparing hand pollinated and open capsules from a given plant (14 low, 10 mid, and eight high elevation, 14 of which came from cool and 18 from the warm glasshouse, respectively). On an additional 22 plants total seed mass (but not number) of hand pollinated versus open-pollinated capsules was assessed (eight low, two mid, and 12 high elevation, 14 of these came from cool and eight from the warm glasshouse, respectively). An Analysis of Variance for Unbalanced designs (growth temperature, elevation and pollination treatment and all interactions included as fixed terms) revealed no significant effect of hand pollination on either seed number per capsule or individual seed mass, regardless of inclusion or otherwise of the growth temperature or elevation terms (results not shown). Thus, we conclude that *W. ceracea* is autogamous and that capsule and seed production in the glasshouse is not likely to be limited by pollen. Hand- and open-pollinated capsules were therefore combined for all subsequent analyses and total number of capsules produced on each plant was used as a proxy for fitness.

## Appendix A3

**Table A3:** Probabilities from REML analysis of emergence and reproductive traits. Values significant at *P* < 0.05 are shown in bold

Source	n.d.f.	Days to emergence (ln)	Flowering date	Capsule number (ln)	Individual capsule mass	Mg seed/capsule	Total capsule mass
Growth Temperature	1	**<0.001**	**<0.001**	**<0.001**	**0.004**	0.898	**<0.001**
Block	4	0.375	0.116	0.156	0.644	0.181	0.153
Growth Temperature × Block	4	0.137	0.174	**0.014**	0.318	0.122	**0.001**
Elevation	2	0.535	0.509	**0.004**	**<0.001**	0.53	0.064
Elevation × Family line	12	**0.019**	0.128	**0.031**	0.573	0.483	**0.045**
Growth Temperature × Elevation	2	0.249	0.229	0.551	0.017	0.336	0.612
Growth Temperature × Block × Elevation	12	0.08	0.629	0.132	0.261	0.059	0.536

## Appendix A4

**Table A4:** Probability values from REML analysis of growth parameters, (A) height, (B) diameter of rosette, and (C) leaf number at 8, 11, and 14 weeks after the first seedlings had emerged and at a standard development time: 90 days. Height, leaf number, and diameter data were log transformed. Juvenile growth increment is calculated from values at 8 and 11 weeks, mature growth increment from 11 and 14 weeks. The increments on mature growth for diameter and leaves required (log) transformation. Bolded values are significant at *P* = 0.05

		8 weeks	11 weeks	14 weeks	Juvenile growth increment	Mature growth increment	90 days
Parameter	df	*P*	*P*	*P*	*P*	*P*	*P*
(A) Height							
Growth Temperature	1	**<0.001**	**<0.001**	**<0.001**	**<0.001**	**<0.001**	**<0.001**
Block	4	0.307	0.556	0.203	0.600	0.021	0.094
Growth Temperature × Block	4	**0.001**	**0.001**	**0.023**	**0.054**	0.092	0.074
Elevation	2	**0.033**	**0.009**	**0.034**	0.225	0.303	0.074
Elevation × Family line	12	0.775	0.137	0.157	0.093	0.795	**0.028**
Growth Temp. × Elev.	2	0.179	0.572	0.475	0.465	0.730	0.414
Growth Temp. × Block × Elev.	12	0.787	0.280	0.587	0.158	0.317	0.268
(B) Rosette Diameter							
Growth Temperature	1	0.063	**0.013**	**0.005**	0.133	0.308	**0.003**
Block	4	0.460	0.344	0.222	0.405	0.659	0.151
Growth Temperature × Block	4	**0.001**	0.093	0.169	**0.009**	0.310	0.070
Elevation	2	**0.047**	**0.036**	0.107	0.810	0.513	0.152
Elevation × Family line	12	0.211	0.162	0.114	0.417	0.065	0.121
Growth Temp. × Elev.	2	0.561	0.745	0.884	0.909	0.362	0.690
Growth Temp. × Block × Elev.	12	0.540	0.549	0.765	0.339	0.869	0.904
(C) Leaf number							
Growth Temperature	1	**0.006**	**0.011**	0.075	0.367	0.091	0.161
Block	4	0.736	0.427	0.12	0.069	**0.028**	**0.049**
Growth Temperature × Block	4	**<0.001**	**0.071**	**0.06**	**0.045**	0.543	0.118
Elevation	2	**0.007**	**0.011**	**0.007**	0.496	0.073	0.076
Elevation × Family line	12	0.657	0.211	0.203	0.077	0.706	0.253
Growth Temp. × Elev.	2	0.666	0.906	0.646	0.893	0.387	0.772
Growth Temp. × Block × Elev.	12	0.617	0.59	0.627	0.386	0.359	0.658

## Appendix A5

**Table A5:** Effects of elevation, maternal line, and treatment on the proportion of methylated MS-AFLP loci. *P* values in the Type III generalized linear model analysis are from Likelihood ratio tests. Bolded values are significant at *P* = 0.05

Source	df	Chi-square	*P* value
Elevation	1	0.30	0.583
Maternal line (Elevation)	8	5.58	0.694
Temperature	1	2.75	0.097
Temperature × Elevation	1	2.17	0.140
Temperature × Maternal line (Elevation)	8	16.23	**0.039**
